# Supplementary material for: Schistosoma mansoni soluble egg antigen and its key proteins differentially affect dextran sodium sulphate-induced inflammatory bowel disease
Source: Mem Inst Oswaldo Cruz. 2026 Mar 2;121:e250243. doi: 10.1590/0074-02760250243 (PMC12952798; doi:10.1590/0074-02760250243)
Supplement: Supplementary material [file 1678-8060-mioc-121-e250243-s1.pdf]

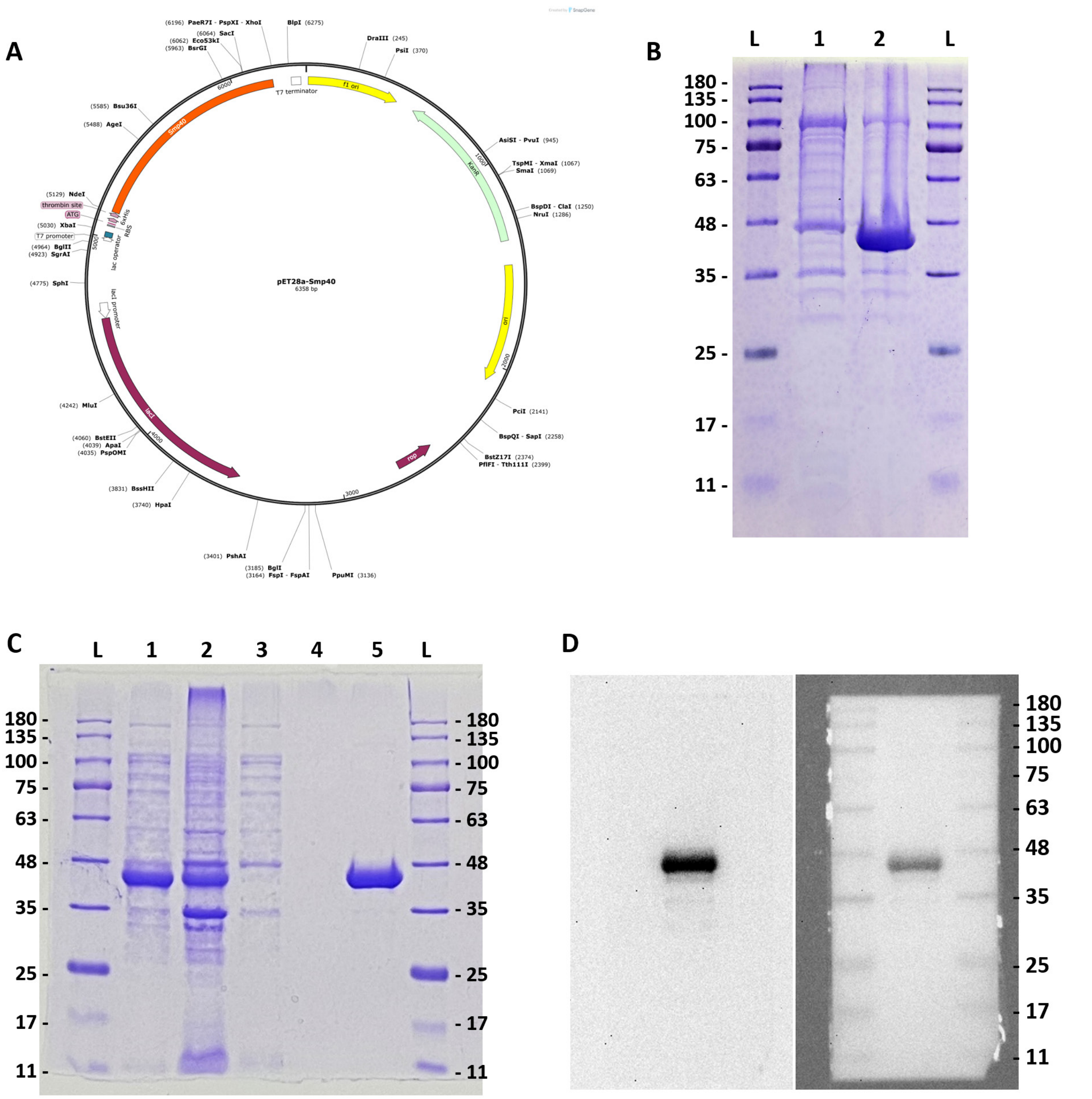

Characterisation of the newly designed Smp40 protein used in this study. (A) Schematic representation of the plasmid construct encoding Smp40. (B) Sodium dodecyl sulphate-polyacrylamide gel (SDS-PAGE) analysis of bacterial lysates with or without IPTG induction. L: protein ladder; lane 1: lysate without IPTG induction; lane 2: lysate after 4 h of IPTG induction. (C) SDS-PAGE analysis of protein purification steps. L: protein ladder; lane 1: supernatant fraction; lane 2: debris fraction; lane 3: flow-through fraction; lane 4: wash fraction; lane 5: eluted protein. (D) Western blot analysis detecting the histidine tag on the purified Smp40 protein.

TABLE I
Scoring system for the disease activity index (DAI)

Score / Parameters	Percentage of weight loss	Presence of rectal bleeding	Stool consistency
0	< 1.0	No bleeding	Normal stool
1	1.1 - 5.0		
2	5.1 - 10.0	Occult blood*	Loose stool
3	10.1 - 20.0		
4	> 20.1	Macroscopic bleeding	Diarrheic stool

*Occult blood was analysed by the Guaiac method; the development of blue colour is considered positive.

TABLE II
Primer pairs used in this study

Species	Gene name	Primer pairs (5' - 3')	Genbank accession
Mus musculus	*β-actin*	Forward CATCCGTAAAGACCTCTATGCC	NM_007393
		Reverse ATGGAGCCACCGATCCACA	
Mus musculus	*IL-2*	Forward CCTGAGCAGGATGGAGAATTACA	
		Reverse TCCAGAACATGCCGCAGAG	NM_008366
Mus musculus	*IFN-γ*	Forward ACTCAAGTGGCATAGATGTGGAAG	
		Reverse GACGCTTATGTTGTTGCTGATGG	NM_008337
Mus musculus	*IL-4*	Forward CTTCCAAGGTGCTTCGCATA	NM_021283
		Reverse CTTATCGATGAATCCAGGCAT	
Mus musculus	*IL-5*	Forward TCAGGGGCTAGACATACTGAAG	NM_010558
		Reverse CCAAGGAACTCTTGCAGGTAAT	
Mus musculus	*IL-17A*	Forward TCAGCGTGTCCAAACACTGAG	NM_010548
		Reverse CGCCAAGGGAGTTAAAGACTT	
Mus musculus	*IL-22*	Forward ATGAGTTTTTCCCTTATGGGGAC	NM_016971
		Reverse GCTGGAAGTTGGACACCTCAA	
Mus musculus	*IL-10*	Forward GGTTGCCAAGCCTTATCGGA	NM_010548
		Reverse ACCTGCTCCACTGCCTTGCT	
